# 1α,25-Dihydroxyvitamin D3 Induces Neutrophil Apoptosis through the p38 MAPK Signaling Pathway in Chronic Obstructive Pulmonary Disease Patients

**DOI:** 10.1371/journal.pone.0120515

**Published:** 2015-04-23

**Authors:** Haihua Yang, Feng Long, Youzhi Zhang, Ronghuan Yu, Peng Zhang, Wenjing Li, Shuijun Li, Xianqiao Jin, Jingwen Xia, Liang Dong, Ning Zhu, Ying Huang, Yi Gong, Xiaodong Chen

**Affiliations:** 1 Department of Respiratory Medicine, Huashan Hospital North, Fudan University, ShangHai, China; 2 Department of Respiratory Medicine, Xuhui Central Hospital, ShangHai, China; 3 Department of Central Laboratory, Xuhui Central Hospital, ShangHai, China; 4 Department of Respiratory Medicine, Huashan Hospital, Fudan University, ShangHai, China; University of Dundee, UNITED KINGDOM

## Abstract

**Background:**

Reduced neutrophil apoptosis plays an important role in the pathogenesis of acute exacerbation chronic obstructive pulmonary disease (AECOPD). The p38 mitogen-activated protein kinase (MAPK) signaling pathway is involved in neutrophil apoptosis. 1α,25-Dihydroxyvitamin D3 (1α,25VitD3) can induce tumor cell apoptosis. The aim of this study was to assess the effects of 1α,25VitD3 on peripheral blood neutrophil apoptosis in AECOPD and examine the role of the p38 MAPK signaling pathway.

**Methods:**

The study enrolled 36 AECOPD patients and 36 healthy volunteers. Venous blood samples were obtained from both groups. Serum 25-hydroxyvitamin D (25-(OH) D) levels in peripheral venous blood were assayed using liquid chromatography-tandem mass spectrometry (LC-MS/MS); the neutrophils were separated and cultured with SB203580 (a p38 inhibitor) and 1α,25VitD3. Neutrophil apoptosis was measured using flow cytometry, and phospho-p38 MAPK protein expression was detected by Western blot. Statistical analysis was performed using analysis of variance. Student's t-test and Pearson's correlation coefficient were used for the between-group differences and correlation analysis, respectively.

**Results:**

The 25-(OH) D levels were lower in AECOPD patients than in healthy controls, and the peripheral blood neutrophil apoptosis results were similar. 1α,25VitD3 increased the apoptosis rate and the level of phospho-p38 MAPK in peripheral blood neutrophils of AECOPD patients. SB203580 partly inhibited 1α,25VitD3-induced peripheral blood neutrophil apoptosis and phospho-p38 MAPK overexpression. The 25-(OH) D levels were positively correlated with increased peripheral blood neutrophil apoptosis and phospho-p38 MAPK levels. In addition, expression of the phospho-p38 MAPK protein was also positively correlated with peripheral blood neutrophil apoptosis.

**Conclusion:**

Our results suggest that 1α,25VitD3 induces peripheral blood neutrophil apoptosis through the p38 MAPK signaling pathway in AECOPD patients.

## Introduction

Chronic obstructive pulmonary disease (COPD), a major cause of morbidity and mortality throughout the world, is characterized by persistent airflow limitation and airway and systemic inflammation responses [1]. The increased number of neutrophils in the lungs and the increased activation and priming of neutrophils in peripheral blood play important roles in the pathogenesis of AECOPD [2–5]. Apoptosis, known as programmed cell death, plays a critical role in regulating the inflammation response. Neutrophil activation by inflammatory stimuli delays apoptosis, resulting in the increased release of inflammatory cytokines that may lead to an inflammatory pulmonary disease with systemic impact, which is related to the course of AECOPD [5,6]. Thus, new therapeutic COPD treatments that focus on resolving neutrophil apoptosis are being explored. Increasing evidence indicates that the p38 MAPK signaling pathway plays an important role in COPD pathogenesis, including chronic inflammation, cell chemotaxis, airway wall remodeling, corticosteroid insensitivity, and airflow obstruction [7–9]. However, the correlation between the P38 MAPK signaling pathway and the apoptosis of peripheral blood neutrophils in COPD is insufficient. Therefore, further studies focusing on the p38 MAPK signaling pathway and peripheral blood neutrophil apoptosis in COPD are necessary.

Recent studies have shown that a substantial proportion of COPD patients have a vitamin D deficiency [10,11], which has been linked to an accelerated decline in lung function, reduced immunity, and increased inflammation in COPD [12–14]. The biological effects of 1α,25VitD3 are primarily mediated via the nuclear transcription factor, vitamin D receptor (VDR), which triggers the expression of vitamin D-responsive genes. Vitamin D and its active form (1α,25VitD3) exert multiple (e.g., apoptosis induction, anti-proliferative) effects in immune regulatory cells and cancer cells [15–18]. Vitamin D receptors are expressed in neutrophils [19]. Hirsch et al. reported that vitamin D down regulates neutrophil function and decreases neutrophil activity, but little is known about the relationship between 1α,25VitD3 and peripheral blood neutrophil apoptosis in COPD [20]. As a result, research on the molecular mechanisms behind the induction of peripheral blood neutrophil apoptosis by 1α,25VitD3 in COPD is necessary.

Therefore, we investigated the effect of 1α,25VitD3-induced neutrophil apoptosis in peripheral blood through the p38 MAPK signaling pathway in COPD patients.

## Methods

### Patients and methods

#### Study subjects

Hospitalized patients with documented COPD diagnoses according to the Global Initiative for Chronic Obstructive Lung Disease (GOLD) criteria and that presented with an acute exacerbation were included. Acute exacerbation was defined by any combination of the following criteria: worsening dyspnea, increase in sputum volume and increase in sputum purulence [21]. All AECOPD patients with a new chest CT scan. Rules inhaled Salmeterol and fluticasone (250 μg) two times a day in patients with COPD. Except for individual cases of oral contraceptive use, no medication was taken by the group subjects. Subjects were excluded if there was a recent history of oral vitamin D and vitamin D intoxication (because of unusual dietary supplementation), HIV infection, immunosuppressive therapy, liver cirrhosis, hypercalcemia, renal insufficiency, chronic enteritis, or diabetes. Asthma, tuberculosis, bronchiectasis, lung cancer, and other lung disorders were also excluded. The study was approved by the Ethics Committee of Huashan Hospital at Fudan University. All participants provided written informed consent.

#### Study design

To investigate the effects of 1α,25VitD3 on the apoptotic behavior of peripheral blood neutrophils in AECOPD patients, 36 AECOPD patients and 36 healthy adult volunteers were enrolled throughout a single year (2 GOLD level III patients, 1 GOLD level IV COPD patient, and 3 healthy controls each month). All patients were investigated upon admission. Peripheral venous blood samples of 25 mL were obtained from the healthy controls and AECOPD patients, Venous blood samples of 5 mL were obtained to detect 25-(OH) D levels, and venous heparin blood samples of 20 mL were obtained and immediately processed for cell culture. On the first day of admission, venous blood was obtained from all AECOPD patients before the administration of drugs. After 3 to 5 days and at discharge (usually after 11 days), demographic and clinical data, lung function tests, smoking habits, and microbiological findings were collected from the patient’s chart. For hospital stays < 11 days, an ambulatory visit was scheduled.

#### Neutrophil separation and culture

Neutrophils were isolated by Percoll density gradient centrifugation (Pharmacia Corporation, US). All neutrophil samples with > 94% viability and purity were confirmed by Trypan Blue staining. The neutrophils were washed and resuspended at a concentration of 1 × 10^6^ cells/mL in RPMI 1640 medium containing 10% fetal calf serum in 5% CO_2_ and incubated at 37°C with 90% relative humidity.

#### 1α,25VitD3 and SB203580 treatment

All neutrophils were isolated from the healthy controls (Group A) and COPD patients. To pretreat the neutrophils from COPD patients in the preliminary experiments, 1 × 10^–7^, 3 × 10^–7^, and 6 × 10^–7^ M doses of 1α,25VitD3 were used, and a dose of 6 × 10^–7^ M 1α,25VitD3 was chosen for additional experiments. Neutrophils from the COPD patients were divided into 4 groups: 1) the COPD control group (Group B); 2) the SB203580(+) + 1α,25VitD3 COPD group (Group C) (Tocris Bioscience, Ellisville, MO), which was pretreated with 2.5 μM × 10^–5^ SB203580 for 0.5 h, followed by incubation with 1α,25VitD3 (6 × 10^–7^ M) (Sigma-Aldrich); 3) the 1α,25VitD3 group (Group D), which was treated with 6 × 10^–7^ M 1α,25VitD3; 4) the COPD SB203580(+) group (Group E), which was pretreated with 2.5 μM × 10^–5^ SB203580 as described for group 2. The abovementioned groups of neutrophils were cultured for 24 h at 37°C. The SB203580 (an inhibitor highly specific for p38) doses were chosen based on previous studies and preliminary experiments. Each group was divided into 2 wells, 1 for flow cytometry and another for Western blot analysis. After the 24 h treatment, neutrophil samples were collected for subsequent experiments.

#### LC-MS/MS method for serum 25-(OH) D

Serum sample was extracted by adding 1 mL of methyl tert-butyl ether and 20 μL of isotope-labeled internal standard to 200 μL of serum. The mixture was vortexed, centrifuged, dried down and then reconstituted in 100 μL of 80% methanol. Serum 25-(OH) D2 and 25-(OH) D3 were determined using a LC-MS/MS method. LC analysis was performed on a Shimadzu system (Kyoto, Japan). Chromatographic separation was achieved on a reverse phase column (50 mm × 4.6 mm, 5 μm, Phenomenex) at a 1.0 mL/min flow rate with a gradient elution using water and methanol (containing 0.2% formic acid) as the mobile phase. An API4000 tandem mass spectrometer (Applied Biosystems/MDS Sciex, Toronto, Canada) equipped with an atmospheric pressure chemical ionization source was used for quantitative analysis.

#### Measurement of the neutrophil apoptotic index by flow cytometry

The percentages of apoptotic neutrophils were determined using the Annexin V-fluorescein isothiocyanate (FITC)/propidium iodide (PI) kit (BioVision) and detected by flow cytometry according to the manufacturer’s protocol. Briefly, after the SB203580 and 1α,25VitD3 treatments, the neutrophils were collected and resuspended in binding buffer ([pH 7.5] 10 mM HEPES, 2.5 mM CaCl_2_, 140 mM NaCl), incubated with Annexin V-FITC/PI for 10 min in the dark, and analyzed using flow cytometry. Neutrophils in the early stage of apoptosis stained positively for Annexin V-FITC; those in the late stage stained positively for both Annexin V-FITC and PI. The data were analyzed using the Modfit and Cell Quest software programs (Becton, Dickinson and Company). Apoptotic neutrophils were measured immediately after obtaining the blood sample (baseline apoptosis) and after culturing for 24 h. The apoptosis rate represents the proportion of apoptotic neutrophils measured minus the baseline value and was considered for further analysis.

#### Western blot detection of phospho-p38 MAPK

The medium was removed, and the cells were washed twice with phosphate-buffered saline (PBS). After adding 0.6mL of cold RIPA buffer (10 mM Tris pH 7.5, 100 mM NaCl, 1 mM EDTA, 0.5% sodium deoxycholate, 0.1% SDS, 1% Triton X 100) and protease inhibitors, the cells were scraped at 4°C. The cell lysate samples were subjected to centrifugation at 14,000 × g for 15min at 4°C. The resultant protein samples were separated by SDS-PAGE and transferred onto a polyvinylidene difluoride membrane (Millipore). The membrane was stained with Ponceau S to confirm the uniform transfer of all samples and then incubated in blocking solution (PBS with 0.05% tween 20 and 5% non-fat dry milk) for 1 h at room temperature. These samples were then incubated overnight with anti-p38 MAPK and anti-phospho-p38 MAPK antibodies (1:1,000 final dilution; Cell Signaling Technology), with anti-glyceraldehyde-3-phosphate dehydrogenase (GAPDH) antibodies (Sigma-Aldrich) as the loading control. Immunoreactive bands were detected by incubation with a horseradish peroxidase–conjugated secondary antibody and a chemiluminescence substrate (Chemicon, Temecula, CA). The scanned films were densitometrically analyzed using Quantity One software (Fuji2000).

### Statistical analysis

The results are expressed as the mean ± standard deviation (SD). The statistical significance between the different groups was determined by analysis of variance (ANOVA) and Student’s t-test; Chi-square statistics were used to analyze participants’ gender and smoking habits. We used the Pearson's correlation coefficient for the correlation analysis. p < 0.05 was considered statistically significant.

## Results

A total of 36 AECOPD patients and 36 healthy adult volunteers were enrolled in the study. The characteristics of the study population are shown in Table 1. Among AECOPD patients, 53% used long-term domiciliary oxygen therapy, and the overall mean FEV_1_ in percent predicted was 39%. There was no significant difference in the gender, age, and smoking habits between AECOPD patients and healthy controls. The 25-(OH) D levels were significantly higher in the healthy controls than in the AECOPD patients.

**Table 1:** Baseline characteristics of the study sample, presented as the mean ± s.d. for continuous variable and as the percentage for categorical variables.

	Controls	AECOPD	*P* **
**Subjects (n)**	36	36	
**Male/Female (n)**	28/8	30/6	0.55
**Age (years)**	70.83±9.3	71.50 ± 8.6	0.95
**Smoking habits (n** ^**#**^ **)**	8/25/3	6/28/2	0.72
**FEV1% pred on admission** *	95±8.5	39±7.4	<0.05
**Resting PaO2†**		8.5±1.8	
**LTDOT %** ^**§**^		53	
**Exacerbations %** ^**##**^			
**<2 last 12 months**		86	
**≥2 last 12 months**		14	
**25-(OH) D levels**	52.3 ± 15.43	33.63± 10.3	<0.05

^#^smokers/ex-smokers/nonsmokers.

*FEV_1_: Forced expiratory volume in 1 s.

†PaO_2_: arterial oxygen tension.

^§^LTDOT: Long-term domiciliary oxygen therapy.

^##^Exacerbations requiring either hospitalization or treatment with oral antibiotics or oral steroids.

**Associations were tested with t-test and Chi-square.

### Effect of 1α,25VitD3 on the apoptosis of neutrophils

**Fig 1:**
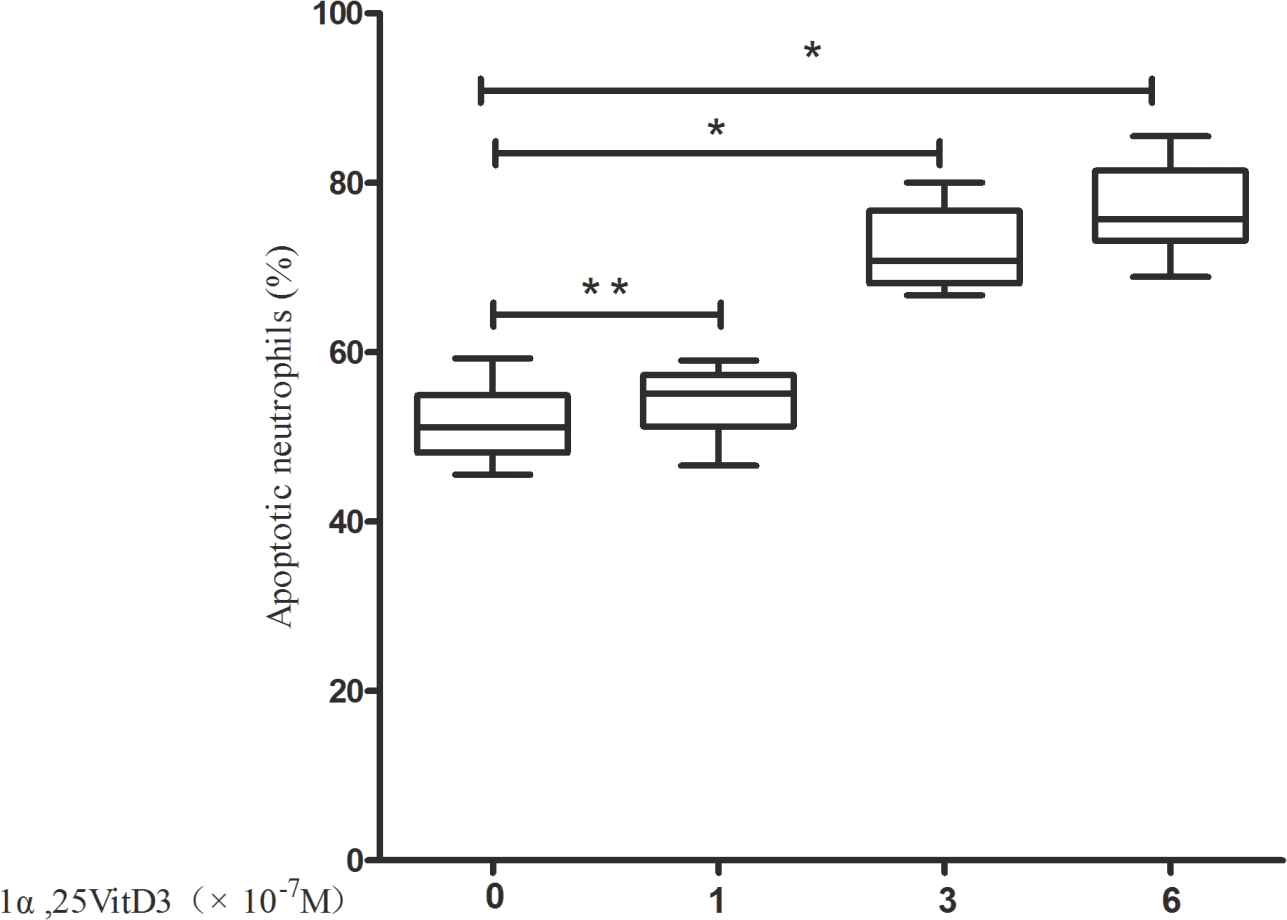
1α,25VitD3 induced peripheral blood neutrophil apoptosis in a dose-dependent manner in the AECOPD patients. Neutrophils were cultured for 24 h in the absence or presence of 1×10^–7^ M, 3 ×10^–7^ M or 6 ×10^–7^ M 1α,25VitD3 as indicated, and then assessed for apoptosis of neutrophils by flow cytometry. The data are presented as the means±sd, n = 6, *P < 0.05, **P > 0.05.

**Fig 2:**
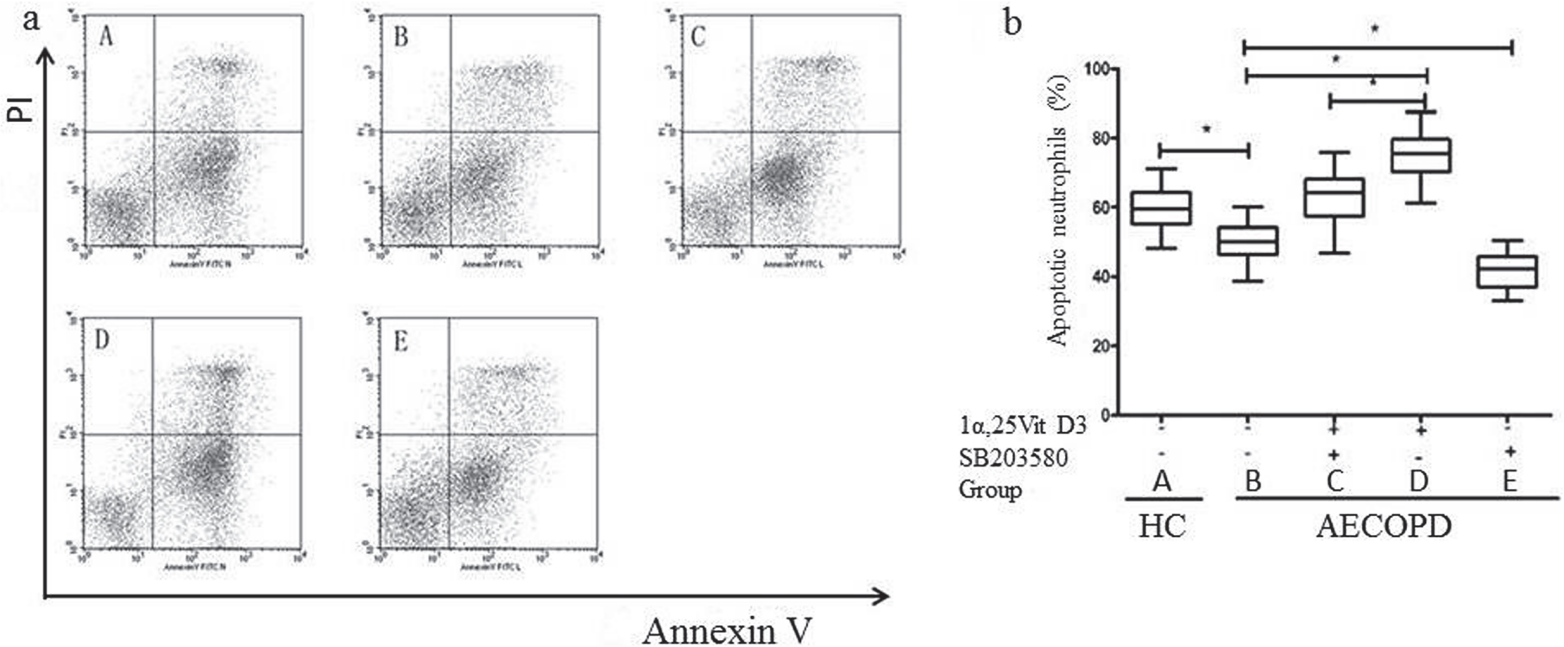
1α,25VitD3 increased peripheral blood neutrophil apoptosis in the AECOPD patients. Neutrophils (1 ×10^6^) were pretreated with SB203580 (2.5 μM × 10^–5^) for 0.5 h, followed by incubation with 1α,25VitD3 (6 ×10^–7^ M) for 24 h. Neutrophil apoptosis was analyzed with flow cytometry (a). The right lower quadrant (RLQ) contains early apoptotic neutrophils with positive Annexin V-PE binding and negative 7-AAD binding (Annexin V-PE [z], 7-AAD [–]). The vital neutrophils in the lower left quadrant (LLQ) are Annexin V-PE (-),7-AAD (-). The upper right quadrant (RUQ) contains late apoptotic and necrotic neutrophils (Annexin V-PE [z], 7-AAD [z]). The amount of neutrophil apoptosis in the RUD and RLD quadrants are shown in (b), expressed as the % of the total cell count (10,000).The experiments shown are representative of at least 3 experiments. The data are presented as the means ± sd, n = 36,*p < 0.05.

The apoptosis rate was upregulated in a dose-dependent manner in the 1α,25VitD3 AECOPD group. These results are shown in Fig 1. Low doses of 1α,25VitD3 (1 × 10^–7^ M) did not significantly affect apoptosis (p > 0.05), However, at higher doses (i.e., 3 × 10^–7^ M and 6 × 10^–7^ M), 1α,25VitD3 increased the apoptosis rate significantly (p < 0.05) in AECOPD patients. Here, we used a 6 × 10^–7^ M dose of 1α,25VitD3 in culture for further investigation. The peripheral blood neutrophil apoptosis rate was increased in the 1α,25VitD3 AECOPD group compared to the AECOPD control group (p < 0.05) (Fig 2).

### Effect of 1α,25VitD3 on the expression of phospho-p38 MAPK

**Fig 3:**
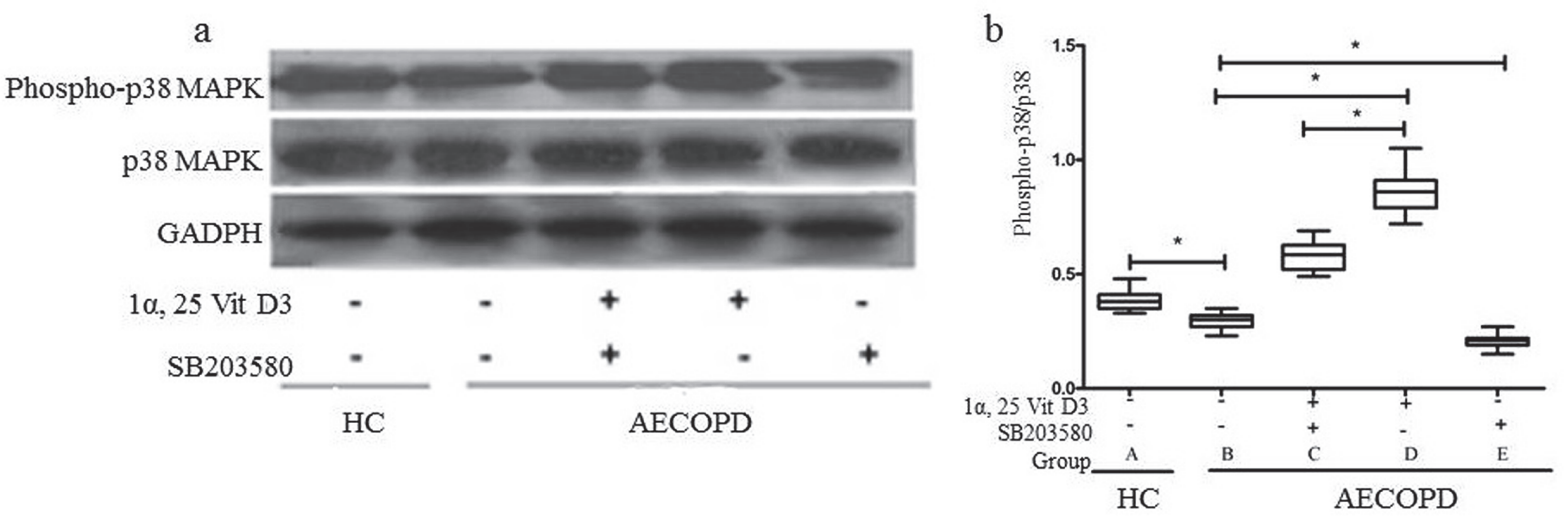
Effect of 1α,25VitD3 on the expression of phospho-p38 MAPK in the peripheral blood neutrophil of the AECOPD patients. Neutrophils (1×10^6^) were pretreated with SB203580 (2.5 μM×10^–5^) for 0.5 h, followed by incubation with 1α,25VitD3 (6 ×10^-7^M) for 24 h. Western blot was used to analyze the phospho-p38 MAPK and p38 MAPK levels. The experiments shown are representative of at least 3 experiments. *p < 0.05.

To assess whether p38 MAPK was activated in the peripheral blood neutrophil apoptosis induced by 1α,25VitD3, we measured the p38 MAPK expression (phospho-p38 MAPK) by Western blot (Fig 3). The phospho-p38 MAPK level was higher in the healthy control group than in the AECOPD control group (p < 0.05). Increased phospho-p38 MAPK levels were detected in the 1α,25VitD3 AECOPD group compared to the AECOPD control group (p < 0.05). Conversely, no differences were observed regarding the total amount of p38 MAPK expressed by neutrophils in all groups (p > 0.05).

### Effect of p38 MAPK inhibitors on 1α,25VitD3-induced neutrophil apoptosis and phospho-p38 MAPK

To further elucidate the role of the p38 MAPK signaling pathway in 1α,25VitD3-induced apoptosis, we observed the effects of SB203580, a specific p38 inhibitor, on 1α,25VitD3-induced apoptosis. As shown in Figs 2 and 3, peripheral blood neutrophil apoptosis was reduced in the SB203580(+) AECOPD group compared to the AECOPD control group (p < 0.05), as was the case with phospho-p38 MAPK protein expression (p < 0.05). Moreover, the peripheral blood neutrophil apoptosis rate was greater in the 1α,25VitD3 AECOPD group than in the SB203580(+) + 1α,25VitD3 AECOPD group (p < 0.05); similar results were observed for phospho-p38 MAPK (p < 0.05).

### Correlative analysis

**Fig 4:**
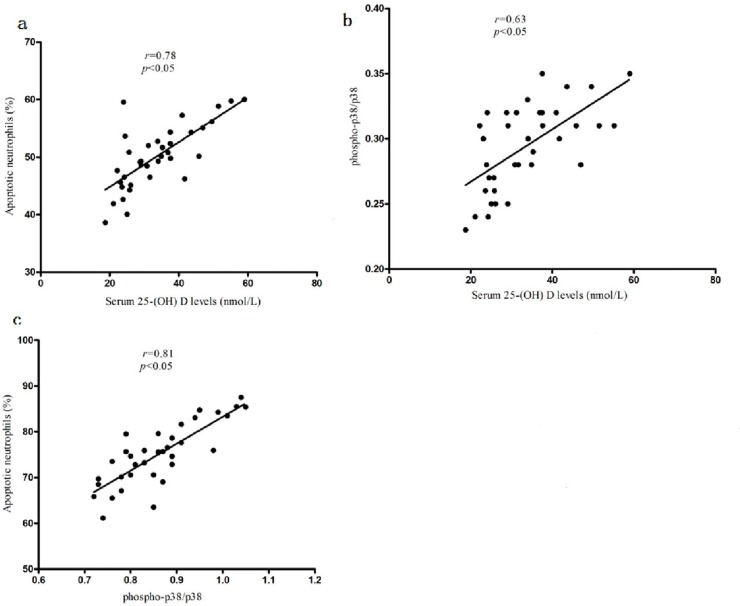
correlation analysis. The serum 25-(OH) D level correlates with the neutrophil apoptosis in the AECOPD patients(a). The serum 25-(OH) D level correlates with the phospho-p38 MAPK in the AECOPD patients(b). The correlation between the neutrophil apoptosis and phospho-p38 MAPK/p38 MAPK in AECOPD patients(c). The *r* and *p* values were assessed using Pearson's correlation analysis.

As shown in Fig 4A and 4B, the 25-(OH) D level was positively correlated between the ratio of peripheral blood neutrophil apoptosis and phospho-p38 protein (r = 0.78 and r = 0.63, respectively, p < 0.05). In addition, there was a positive correlation between the phospho-p38 protein level and the ratio of peripheral blood neutrophil apoptosis (r = 0.81, p < 0.05, Fig 4C).

## Discussion

COPD patients are at particularly high risk for developing neutrophil-mediated inflammatory diseases. It has been assumed that the neutrophil half-life in vitro is a reflection of its life span in vivo [22]. Our experiments found that the peripheral blood neutrophil apoptosis rate of AECOPD patients is lower than that in healthy controls, which may result from the pathogenesis of COPD. Our results are in agreements with a previous report [6].

The possibility that vitamin D insufficiency and deficiency may increase COPD risk has only been recognized recently [11–13]. Serum 25-(OH) D levels reflect vitamin D status. Low 25-(OH) D levels were commonly observed in COPD, which may be due to the diminished production of pre-vitamin D3 associated with skin aging caused by smoking and limited UVB exposure [23,24]. Our experiments found that the rate of vitamin D deficiency or insufficiency in AECOPD patients was high. Meanwhile, another study also observed a high prevalence of vitamin D deficiency, and the relationship between lung function and systemic levels of vitamin D was almost linear [11]. In a recent study using vitamin D as a treatment for COPD, Lehouck et al. [25] found that high doses of vitamin D supplementation significantly reduced the exacerbation rate per patient-year by 43% in COPD patients with severe vitamin D deficiency, However, the underlying mechanisms between 1α,25VitD3 and AECOPD pathogenesis are poorly understood. Notably, this study showed that 1α,25VitD3 induced apoptosis in the neutrophils of AECOPD. In our study, we investigated whether 1α,25VitD3 could induce peripheral blood neutrophil apoptosis in AECOPD in vivo and examined the appropriate concentration of 1α,25VitD3 required for intervention. We found that 1α,25VitD3 could not induce peripheral blood neutrophil apoptosis at a dose of 1 × 10^–7^ M, which was confirmed by data from Hirsch et al. [20]. However, 1α,25VitD3 could significantly induce peripheral blood neutrophil apoptosis in AECOPD patients at 3 × 10^–7^ M and 6 × 10^–7^ M doses, which are consistent with the conditions used in NB4 cell lines [26]. This result supported the hypothesis that 1α,25VitD3 could induce peripheral blood neutrophil apoptosis in AECOPD patients, and the efficiency of apoptosis induction is best at 6 × 10^–7^ M.

The p38 MAPK signaling pathway plays an important role in human neutrophil apoptosis [22]. To explore the possible mechanism of peripheral blood neutrophil apoptosis in AECOPD patients, we used SB203580, a specific p38 MAPK inhibitor, which significantly decreased neutrophil apoptosis. We also observed that the serum25-(OH) D levels were positively correlated with phospho-p38 protein and with peripheral blood neutrophil apoptosis. In addition, the levels of phospho-p38 MAPK and apoptotic neutrophils were lower in AECOPD patients than those in healthy controls. The phospho-p38 MAPK level positively correlated with peripheral blood neutrophil apoptosis. The results indicated that 1α,25VitD3 could induce peripheral blood neutrophil apoptosis in AECOPD via p38 MAPK activation. Therefore, we speculated that vitamin D insufficiency and deficiency may affect the activation of phospho-p38 MAPK and lead to decreased peripheral blood neutrophil apoptosis, which may be one possible important pathogenesis of AECOPD. However, determining whether other apoptotic signaling pathways can affect peripheral blood neutrophil apoptosis in COPD is worthy of further study.

## Conclusion

Our data demonstrated that 1α,25VitD3 could induce peripheral blood neutrophil apoptosis in AECOPD and that the mechanism of apoptosis was related to the p38 MAPK pathway.
